# Explainable Artificial Intelligence (XAI) and Molecular Modeling Techniques to Discover Putative HER2 Inhibitors

**DOI:** 10.3390/ijms27146504

**Published:** 2026-07-22

**Authors:** Shailima Rampogu, Thananjeyan Balasubramaniyam, Cheol-Hee Yoon, Yongseong Kim, Jacek Z. Kubiak, Keun Woo Lee

**Affiliations:** 1 Cachet Big Data Lab, Hyderabad 500081, Telangana, India; shailima.rampogu@gmail.com; 2 Laboratory of Molecular Oncology and Innovative Therapies, Military Institute of Medicine—National Research Institute, Szaserow 128, 04-141 Warszawa, Poland; dhanaj8@gmail.com; 3 Division of Chronic Viral Diseases, Center for Emerging Virus Research, Korea National Institute of Health, 187 Osongsaengmyeong 2-ro, Cheongju 28159, Republic of Korea; kmc755@korea.kr; 4 Department of Pharmaceutical Engineering, Kyungnam University, Changwon 51767, Republic of Korea; kimys@kyungnam.ac.kr; 5 Dynamics and Mechanics of Epithelia Group, Institute of Genetics and Development of Rennes (IGDR), National Centre for Scientific Research (CNRS), Faculty of Medicine, University of Rennes, UMR 6290, 35043 Rennes, France; 6 Korea Quantum Computing (KQC), 55 Centumjungang-ro, Haeundae-Gu, Busan 48058, Republic of Korea; 7 Angel i-Drug Design (AiDD), 33-3 Jinyangho-ro 44, Jinju 52650, Republic of Korea

**Keywords:** HER2, explainable artificial intelligence, molecular fingerprints, natural compounds, classification methods

## Abstract

Breast cancer is one of the prominent reasons of death in women. HER2 is a promising target to counter breast cancer. In the current research, a structure-based pharmacophore model was generated to map and screen CMNPD, a comprehensive database of marine natural products. The two compounds (CMNPD30448 (hit1) and CMNPD7060 (hit2)) displayed better LibDock scores than the reference co-crystallized ligand. These compounds demonstrated stable molecular dynamics results conducted for 500 ns with stable root mean square deviation (RMSD) at 0.3 nm, stable radius of gyration (Rg) and root mean square fluctuation (RMSF). On ChEMBL compounds, different PaDEL descriptors and various machine learning (ML) and neural network (NN) methods were used. The results showed that PubChem fingerprints with random forest classification model displayed an accuracy of 0.91 and a receiver operating characteristic area under the curve (ROC-AUC) of 0.96. This model further predicted the retrieved compounds as ‘active’. The explainable random forest with LIME showed that PubChem fingerprint440 [C(-C)(-O)(=O)], PubChem fingerprint452 [C(-O)(=O)], PubChem fingerprint380 [C(~O)(~O)], PubChem fingerprint566 [O-C-C-N] and PubChem fingerprint712 [C-C(C)-C(C)-C] for hit1 and PubChem fingerprint700 [O-C-C-C-C-C-O-C], PubChem fingerprint380 [C(~O)(~O)], and PubChem fingerprint712 [C-C(C)-C(C)-C] for hit2 have contributed towards plausible inhibitory potential. These findings suggest the two compounds CMNPD30448 and CMNPD7060 might serve as HER2 inhibitors. Further in vitro and in vivo analysis are required before using them.

## 1. Introduction

Human Epidermal Growth Factor Receptor 2 (HER2) is a 185 kDa protein (p^185^) [1] that encompasses an external ligand binding domain [2] and an internal tyrosine kinase domain. This is also referred to as HER2/neu, ErbB2, or c-erbB2. Furthermore, HER1 (ErbB1 or EGFR), HER3 (ErbB3), and HER4 (ErbB4) are the other three structurally similar members of the HER family [3,4]. Because HER2 lacks a known direct soluble ligand, it joins with the other HER members for heterodimer formation and promotes the most effective signal transduction [5,6]. Additionally, in HER2-amplified cells, ligand-independent homodimers are observed [7]. HER2 communicates with cancer cell signaling, thereby promoting proliferation [8]. In the cytoplasmic domain, the formation of dimers promotes tyrosine kinase residues to undergo phosphorylation, which triggers signaling pathways such as mitogen-activated protein kinase (MAPK) and phosphatidylinositol triphosphate kinase (PI3K) that steer the cell cycle progression and proliferation [9].

HER2 demonstrates a crucial role in the growth and differentiation of a normal cell [10]. Under conditions when HER2 gene is amplified, the receptor is correspondingly amplified, causing several cancers, including breast cancer. Breast cancer is widely noticed in women, and the failure of therapeutics is due to resistance to traditional drugs [10]. Overexpression of HER2 is the initial occurrence in the development of breast cancer, noted in 50% of in situ carcinomas [6]. Thus, HER2 can serve as a therapeutic target for the identification of small therapeutic molecules [11,12,13]. In HER2-positive breast cancer, metastases to the brain are frequently observed in 25–50% of women [14,15] with a poor probability of survival [16]. Considerable evidences suggest that both EGFR and HER2 are amplified in breast cancer brain metastases, thereby contributing to the spread of metastatic lesions inside the brain and hence serving as promising targets [15,16].

In recent times, multiple computational methods have been playing a significant role in identifying new and effective agents against various diseases [17,18,19]. Typically referred to as computer-aided drug discovery/design, it has structure-based and ligand-based approaches [20]. Structure-based drug discovery, sometimes called target-based drug discovery, exploits pivotal features between the target’s key residues and the in-bound ligand [21,22]. This method executes the essential stages of drug discovery, such as ‘hit identification’ and ‘hit-to-lead’. The so-obtained hits presumably demonstrate efficacy and affinity towards the target [23]. Structure-based drug design has evolved into a beneficial, affordable, and swift computational strategy for determining potential compounds that correspond to the target’s structure [24].

Machine learning is a subgroup of artificial intelligence through which algorithms can be developed from unrefined data that can be employed for later use. These algorithms implement regression, clustering, and classification models [25]. Typically, fingerprints embed the structural characteristics of a given molecule. These act as feature identities that are displayed as vectors. The individual vector component defines the presence, degree, and probability or frequency of the particular feature. These fingerprints are paramount in the drug discovery mechanism [26]. A study has reported molecular interaction fingerprints to discover GPCR drugs [27]. Another research suggests a Fingerprint-embedding framework for Drug-Target binding Affinity (FingerDTA), an architecture for estimating the affinity of drugs and targets. This approach is built on CNN to retrieve local patterns and uses fingerprints for global information characterization [28]. Furthermore, artificial intelligence and machine learning studies are widely employed to predict the active compounds from the inactive compounds, de novo molecule design [29,30] and to classify the small molecules [31,32,33].

In the current investigation, we have employed pharmacophore-based molecular modeling methods complemented by artificial intelligence and machine learning approaches to retrieve two natural compounds as putative HER2 inhibitors.

## 2. Results

### 2.1. Structure-Based Pharmacophore Generation

**Figure 1:**
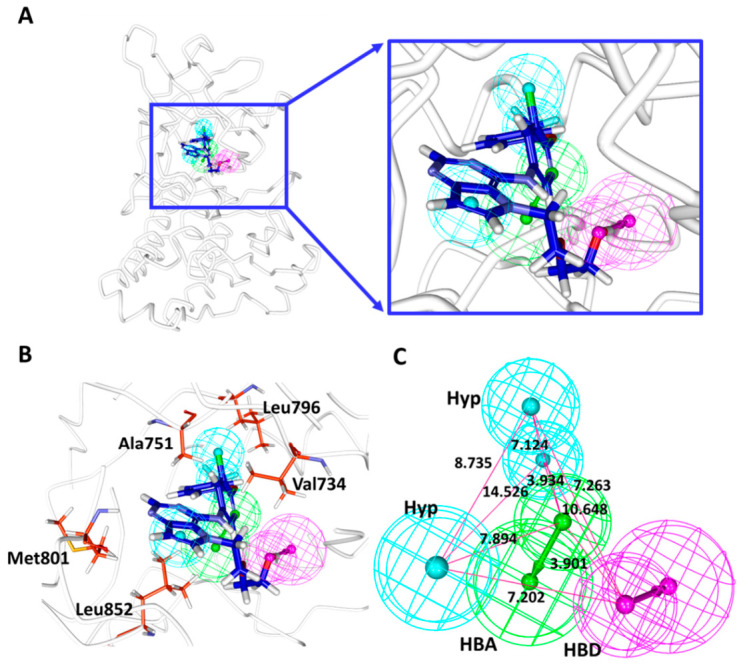
Generation of structure-based pharmacophore model. (**A**) Pharmacophore model aligned to the co-crystallized target structure. (**B**) Key residues complementary to the pharmacophore model. (**C**) Geometry of the pharmacophore model. Abbreviations: HY/HYP, hydrophobic feature; HBA, hydrogen bond acceptor; HBD, hydrogen bond donor; XV, exclusion volume.

A structure-based pharmacophore was built considering the co-crystallized ligand and the target interactions. Correspondingly, a total of 10 pharmacophore models were obtained with five features and the same selectivity score of 9.4812. Furthermore, seven models have produced the same features, while three models differed (Supplementary Table S1). In order to select the best model, a scrupulous examination of the alignment between the pharmacophore features and the key residue interactions was performed. Here, consideration was given to the model that maps with the critical residues that have shown critical interactions, and, hence, model 1 was selected (Figure 1A). This model has been shown to map with Val734, Ala751, Leu796, Met801, and Leu852 (Figure 1B). Besides these, other key residues were also mapped with the model (Supplementary Figure S1). The model has two hydrophobic features, one hydrogen bond acceptor and one hydrogen bond donor (Figure 1C). The selected pharmacophore model was employed for further investigation.

### 2.2. Validation of the Selected Pharmacophore Model

To investigate if the pharmacophore model could retrieve active compounds from a given database (here compounds are obtained from BindingDB, https://www.bindingdb.org/rwd/bind/index.jsp accessed on 12 March 2026 and the literature), the decoy set was instituted, consisting of 1000 (*D*) compounds with 90 active compounds (**A**). Upon mapping with the selected pharmacophore model, a total of 100 (*Ht*) compounds were retrieved, along with 87 active compounds (*Ha*). The EF score and the GH score were calculated to be 9.6 and 0.87, respectively, suggesting that the model is of superior quality.

### 2.3. Virtual Screening for the Retrieval of Drug-like Compounds

The validated pharmacophore model was upgraded to retrieve potential candidate compounds from the larger chemical database. A total of 1192 compounds have satisfied the ADMET properties, while 939 compounds have obeyed Lipinski’s rule. These compounds were escalated to pharmacophore mapping, which resulted in 28 compounds. These 28 compounds were subjected to molecular docking studies to gain insight on their binding affinities and binding mode [34,35,36].

### 2.4. Molecular Docking-Based Screening

**Figure 2:**
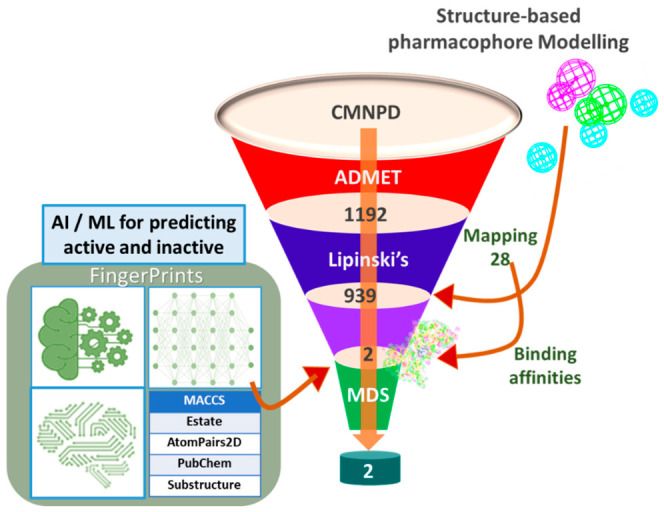
Virtual screening method employed for retrieving potential hits.

The 28 compounds were subjected to binding mode analysis and binding affinity examination [37] to identify potential compounds for effective therapeutics using LibDock [38,39,40]. In the LibDock method, the different ligand conformations associate with the apolar and polar receptor interaction sites called hotspots thereby projecting the pose with the best score [41]. For retrieving the effective compounds, the co-crystallized ligand was considered the reference compound [42]. The results have shown that two compounds have demonstrated a better dock score than the reference compound (Figure 2). The best pose for each compound are selected from the largest cluster that has displayed key residue interactions. The reference compound has displayed a LibDock score of 152.553, while compound CMNPD30448 (hit1) and compound CMNPD7060 (hit2) have shown a binding affinity of 164.72 and 158.332, respectively (Table 1), complemented by key residue interactions. Therefore, these compounds were assessed for bioactivity prediction using fingerprints.

### 2.5. Predicting the Compounds Bioactivity

**Figure 3:**
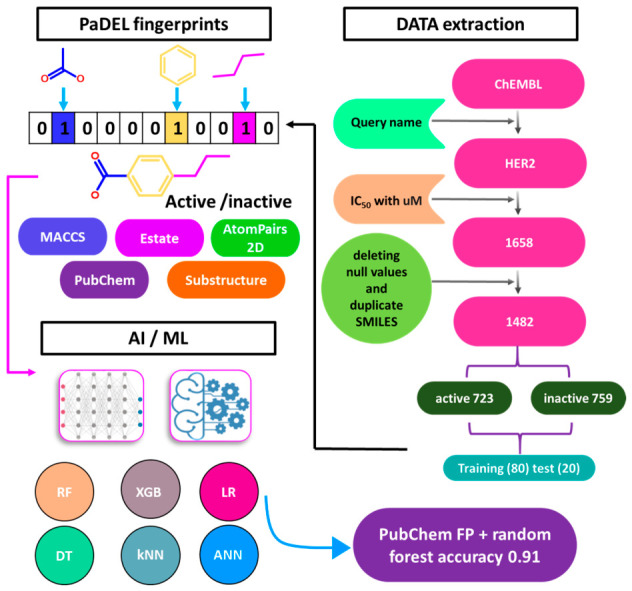
Various steps involved in predicting the classification of potential compounds.

From the ChEMBL database, the IC_50_ with ‘uM’ has resulted in a total of 1658 compounds. Subsequently deleting the ‘null values’ and duplicate ‘SMILES’ filtered the compounds to 1482. These compounds were grouped into active, which resulted in 723 compounds, and inactive, which resulted in 759 compounds. Correspondingly, the PaDEL fingerprints [43] were generated. The PaDEL fingerprints that are employed for the current investigation are MACCS, Estate, AtomPairs2D, PubChem, and Substructure [44]. Furthermore, MACCS [45,46] has 166 bits, Estate [47,48] has 79 bits, AtomPairs2D [48,49] has 780 bits, PubChem [47,48,50] has 881 bits, and Substructure has 307 bits. The classification algorithms were applied employing different methods such as random forest, XGBoost, kNN, logistic regression, decision tree and ANN (Figure 3).

The results have shown that the random forest algorithm demonstrated an overall good accuracy for all the fingerprints, while XGBoost showed a lower accuracy (Table 2). Furthermore, logistic regression, decision trees, ANN, and kNN have shown consistent accuracy. The PubChem fingerprints have displayed better accuracy with all the algorithms (Table 2). From the comprehensive results, it was revealed that PubChem fingerprints with a random forest algorithm showed a greater accuracy of 0.91, and therefore this model was used to predict the bioactivity of the compounds obtained from molecular docking.

The two compounds that have shown a better LibDock score than the reference compound were subjected to AI and ML analysis to predict if these compounds are active, thereby ensuring remarkable therapeutics. The two compounds were upgraded to Google Colab and their PubChem fingerprints were predicted. Furthermore, the random forest model was used, and the compounds were then predicted based upon the PubChem fingerprints (Figure 3). The results have shown that the two compounds were predicted to be active.

#### Evaluation Metrics

The evaluation metrics refers to the capability of the model’s performance. Accordingly, for every FP the evaluation metrics has been performed and the accuracy, precision, recall, F1-score and ROC AUC were recorded (Table 2). The results showed that the RF model has performed well with all the FPs and XGB was the worst performer. The AUC ROC that corresponds to the RF has also shown promising results. Therefore, the RF model is generally regarded as best model in the current study. Upon careful observation the results showed that the RF model with PubChem FP has demonstrated good results with high accuracy (0.91) and high AUC ROC (0.96), (Table 2). Therefore, this model was chosen for further studies.

**Figure 4:**
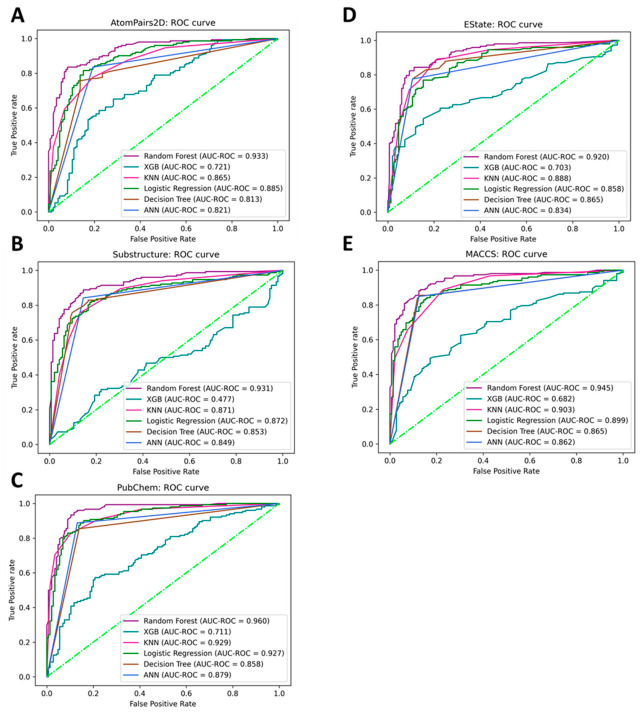
The AUC-ROC of different ML methods for various FPs. (**A**) AUC-ROC for atomPairs2D FP. (**B**) AUC-ROC for Substructure FP. (**C**) AUC-ROC for PubChem FP. (**D**) AUC-ROC for EState FP. (**E**) AUC-ROC for MACCS FP. The dashed diagonal line represents the random-classifier reference (AUC = 0.5)

The ROC graph is one of the versatile methods employed for arranging, visualizing and choosing a classifier according to their performance. This approach was employed in signal detection theory and was also used in understanding the diagnostic systems. The implementation of ROC in ML was performed by Spackman who showed their remarkable significance of comparing and assessing algorithms [51]. Typical ROC plots are 2D graphs consisting of TP rate on the *Y*-axis and FP rate on the *X*-axis and correspondingly describes the trade-offs between true positives and false positives. The findings of the ROC are read as a scalar value called the area under an ROC curve (AUC). Furthermore, given that the AUC is a proportion of the unit square area, the AUC value exists between 0 and 1.0 [51]. This curve acts as a method to determine the effectiveness of the model performance [52] and explicitly renders a model to be good or bad [53]. An AUC value of 1.0 infers a perfect value with no false negatives and no false positives, while an AUC of 0.0 interprets an extremely bad value. Accordingly, AUC between 0.9 and 0.99 represents an excellent value, 0.8 and 0.89 refers to good value, 0.7 and 0.79 indicates a fair value, 0.51 and0.69 implies a poor value, and 0.5 or less denotes an insignificant value [54]. The results have demonstrated that the RF model was the best performer with all the FPs and XGB was the worst performer (Figure 4). For the current study, the probabilities are not considered for neural network approaches. Our results have shown that the RF model with PubChem FP has generated an AUC of 0.96 which is an excellent value (Figure 4). This model was then used to predict the bioactivity of new compounds.

Furthermore, we intended to explain the random forest with Local Interpretable Model-agnostic Explanations (LIME). This is one of the explainable artificial intelligence (XAI) approaches that attempts to provide distinct and valuable support for the predictions rendered by AI and ML. Typically, this method can be adapted for elucidating local explanations where the features that have impacted certain predictions can be determined [55]. The LIME explanation has been widely used in the drug discovery process, though it should be noted that LIME identifies which fingerprint features drive the classifier’s prediction rather than establishing that these features causally mediate physical binding to HER2 [56,57].

**Figure 5:**
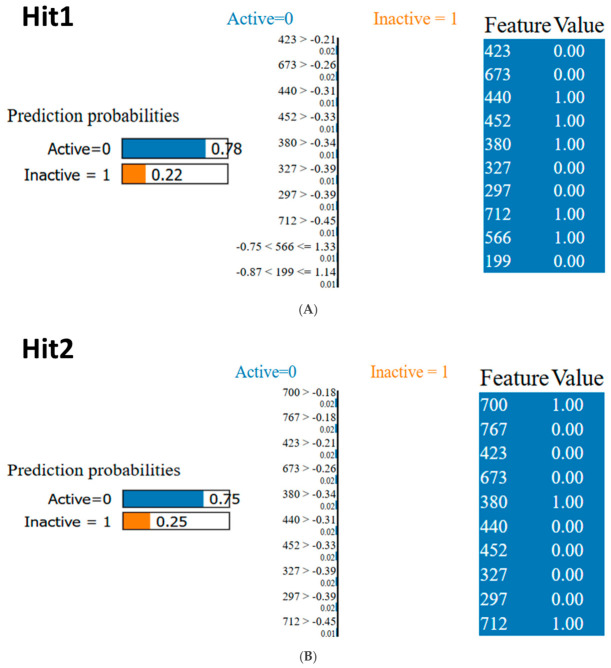
LIME results for hit1 and hit2. (**A**) LIME prediction-probability and feature-contribution plot for hit1 (prediction probability 0.78). (**B**) LIME prediction-probability and feature-contribution plot for hit2 (prediction probability 0.75).

The results showed that hit1 and hit2 demonstrated prediction probabilities of 0.78 and 0.75 (Figure 5A,B), respectively. Particularly for hit1, the major contributing features towards predicting active compounds are PubChem440, PubChem452, PubChem380, PubChem712, and PubChem566, respectively. For hit2 the contributing features are PubChem380, PubChem700 and PubChem712, respectively. Upon viewing the features, it can be observed that two features, PubChem380 and PubChem712, are the major contributors towards predicting the compound to be active (Figure 5). These compounds were then forwarded to MDS to elucidate the nature of the protein–ligand complex at the binding pocket of the target.

### 2.6. Molecular Dynamics Simulations

MDS is a phenomenal approach to probe into the target-ligand complex, particularly with respect to the ligand stability in the binding pocket of the target. Typically, MDS aids in the exploration of highly complex biological systems in motion [58]. In the present investigation, the nature of the complexes are studied by evaluating the root mean square deviation (RMSD), radius of gyration (Rg), root mean square fluctuations (RMSF), exploration of the binding mode, and interactions between the target ligand and hydrogen-bond count. The selected complexes from the molecular docking were subjected to MDS for 500 ns for the protein backbone.

#### 2.6.1. The Protein Backbones Were Stable During the Simulation Run

**Figure 6:**
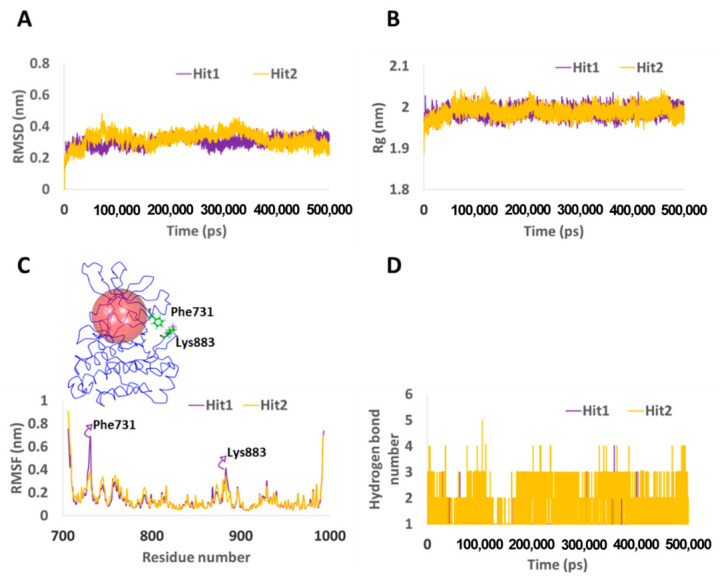
Molecular dynamics simulation results for 500 ns. (**A**) RMSD for backbone atoms of hit1 and hit2. (**B**) Compactness assessment with radius of gyration. (**C**) Fluctuation analysis for hit1 and hit2. Two residues of hit1 demonstrated fluctuations that are away from the active site. (**D**) Hydrogen bond number during 500 ns simulation run.

The RMSD imparts knowledge on the deviations if any that exist between the initial and the final structures correspondingly implying that the smaller the deviation, the greater the stability [59]. Largely, both the complexes have exhibited stable RMSD values within the acceptable range of 0.3 nm. Upon meticulous and in-depth inspection, it was observed that hit1 demonstrated two minor deviations, one at 156,290 ps and another at 273,440 ps. After the second dip the plot seems to be principally stable without any deviations (Figure 6A). Similarly, hit2 displayed dips at 162,360 and 380,730 and remained stable thereafter. Overall, both the compounds have displayed stable profiles with an average RMSD of 0.3 nm each (Figure 6A).

#### 2.6.2. MDS Showed That the Protein Backbones Were Compact

The Rg plots interpret the compactness of the protein structure [60]. The Rg profiles of hit1 and hit2 have shown high compactness (Figure 6B), existing between 1.9 nm and 2 nm. The average of hit1 and hit2 was observed to be 1.98 nm, implying that the systems were highly compact during the simulation run.

#### 2.6.3. Fluctuation Analysis During MDS Analysis

The RSMF calculations evaluate an atom’s or a group of atoms’ dislocation with respect to the reference structure [58]. These plots are important in understanding the fluctuations with respect to each residue [61] during the evolution of the MDS [41]. The results revealed that hit2 did not show any abnormal fluctuations and was remarkably stable (Figure 6C). On the contrary, hit1 showed fluctuation with two residues, Phe731 and Lys883 (Figure 6C). Although Phe731 has demonstrated a larger fluctuation than Lys883, these exist away from the binding pocket and may have a limited potential to influence the binding prospective [59].

#### 2.6.4. Hydrogen-Bond Count During MDS

The occurrence of hydrogen bonds was carefully tracked across the simulation. This analysis may also help in understanding if the prospective compound has occupied the binding pocket while the MDS is progressing. In addition to hydrogen bonds, other key residues may also interact with the ligand to position it in the binding pocket. The results have displayed that the hits have represented hydrogen bond interactions throughout the simulation run. Upon comparison, hit2 rendered more hydrogen bonds than hit1, with an average of 0.62 for hit1 and 1.34 for hit2. Furthermore, the average hydrogen bond number is observed to be greater in the last 100 ns, with an average of 0.79 for hit1 and 0.94 for hit2, respectively (Figure 6D).

#### 2.6.5. Binding Mode Analysis

**Figure 7:**
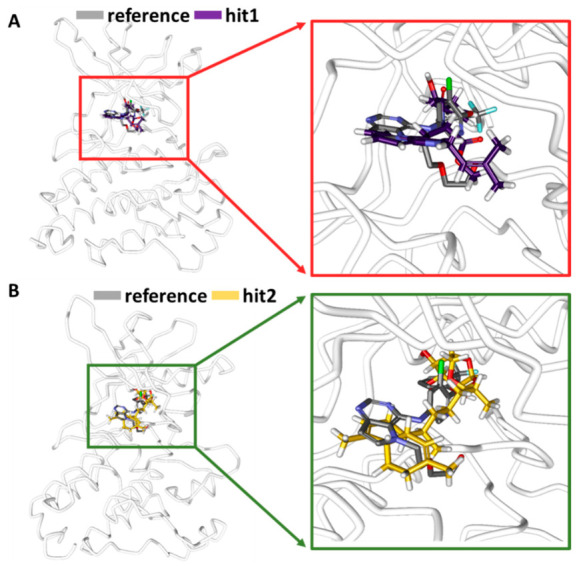
Binding mode analysis of hit1 and hit2. (**A**) The hit1 has accommodated in a similar pattern as that of the co-crystallized ligand. (**B**) The hit2 has accommodated in a similar pattern as that of the co-crystallized ligand. Different colors distinguish the reference ligand, hit1, and hit2.

In order to probe into the binding mode of the ligand, the representative structure was extracted from the stable RMSD of the last 50,000 ns. These structures were aligned onto the X-ray crystal structure to comprehend the binding mode, enabling the Align Structures tool, available with Discovery Studio (DS; BIOVIA Discovery Studio, Discovery Studio v19, Dassault Systemes, San Diego, CA, USA), with a C-alpha distance cutoff of 2.5. Correspondingly, it was observed that both hits have occupied the binding pocket similar to that of the co-crystallized ligand (Figure 7A,B) implying that the identified hits may impart effective therapeutics like that of the co-crystallized ligand. In order to help the *hits* fit into the binding pocket, several residues from the target were clamped to the small molecules.

#### 2.6.6. Detailed Analysis of Intermolecular Interactions

1. *Hit1*

**Figure 8:**
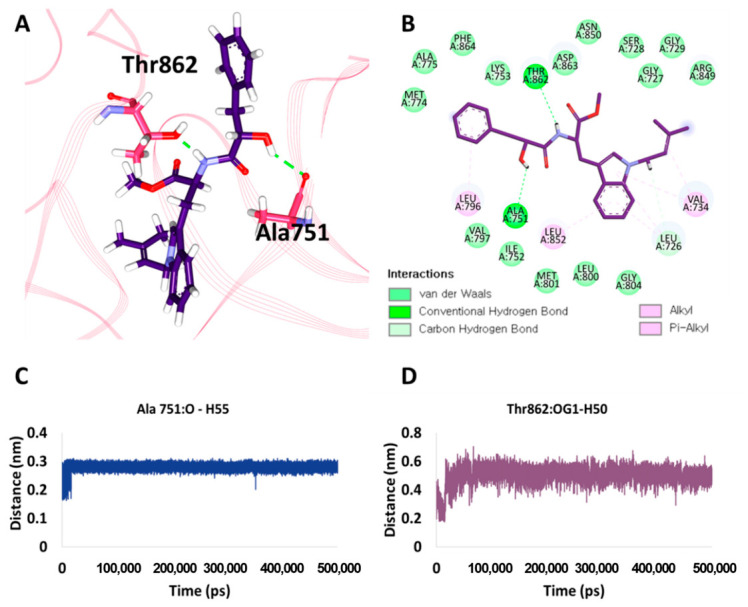
Intermolecular interaction between hit1 and the target. (**A**) The hydrogen bond interaction of hit1 with the target residues. (**B**) Comprehensive intermolecular interaction of hit1 and target residues. (**C**) Hydrogen bond distance between the residue atoms Ala751:O and ligand atom H55. (**D**) Hydrogen bond distance between the residue Thr862:OG1 and ligand atom H50.

We further advanced to look into the comprehensive intermolecular interactions between the small molecules and the target. Hit1 has generated two hydrogen bonds with the key residues Ala751 and Thr862 (Figure 8A,B), respectively. The O atom of Ala751 has interacted with the H55 atom (Figure 8C) of the ligand, and the OG1 atom of the residue Thr862 has formed a bond with H50 of the ligand (Figure 8D). The molecular docking results have also shown interaction with these residues, in which Ala751 has formed a π-alkyl interaction while Thr862 demonstrated a hydrogen bond. However, when the distance measure was conducted during the MDS, it was observed that Thr862 demonstrated an average distance of approximately 0.5 nm; given that this distance exceeds typical donor–acceptor cutoffs for a classical hydrogen bond, this interaction is better described as a weak/long-range polar contact rather than a stable hydrogen bond. Interestingly, another key hydrogen bond interaction with Ala751 has demonstrated an average distance of 0.28 nm throughout the MDS, holding the ligand firmly in the binding pocket.

Ala751 adhered to the small molecule via alkyl and a π-alkyl interaction in the X-ray structure. The residue Thr862 has a π-donor hydrogen bond in the X-ray structure and a hydrogen bond in the molecular docking. The important residue Leu726 has contributed a carbon–hydrogen bond that formed a π-alkyl interaction during the molecular docking and a π-sigma bond in the X-ray structure. The residues Leu726, Val734, Leu796, and Leu852 exhibited alkyl and π-alkyl interactions (Table 3). The π-alkyl interaction of Leu726 appeared to be preserved. Furthermore, Gly727, Ser728, Gly729, Ile752, Lys753, Met774, Ala775, Val797, Leu800, Met801, Gly804, Arg849, Asn850, Asp863, and Phe864 have generated van der Waals interactions positioning the ligand at the binding pocket (Table 3 and Figure 8B).

2. *Hit2*

**Figure 9:**
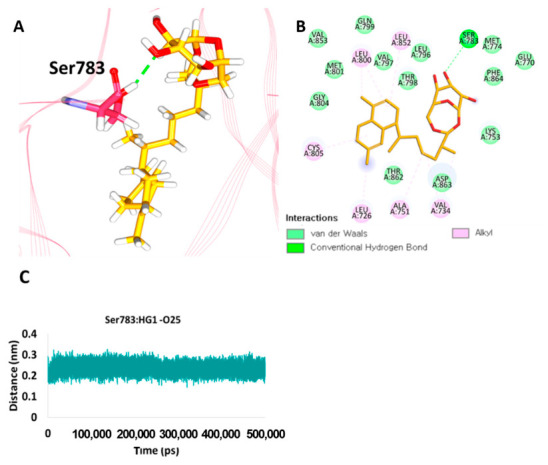
Intermolecular interaction between hit2 and the target. (**A**) The hydrogen bond interaction of hit1 with the target residues. (**B**) Comprehensive intermolecular interaction of hit2 and target residues. (**C**) Hydrogen bond distance between the residue atoms Ser783: HG1 and ligand atom O25.

Hit2 formed a hydrogen bond interaction with the key residue Ser783 (Figure 9A,B). The HG1 atom of Ser783 interacts with O25 (Figure 9C). This residue has also generated a hydrogen bond in the molecular docking that was preserved during the MDS. The residue Ser783 formed a van der Waals interaction in the X-ray structure. Furthermore, molecular docking results have also shown Thr798 and Asp863, which have prompted van der Waals interaction after the MDS. Upon further probing into the distance of the hydrogen bond of Ser783 was observed to be consistent throughout the MDS, with an average of 0.23 nm, implying that the bond is strong and accommodates the ligand at the binding pocket (Figure 9C). Another observation is that Leu800 formed an alkyl interaction in the MDS and the molecular docking result, while it generated a van der Waals interaction in the X-ray structure. Additionally, Leu726, Val734, Ala751, Cys805, and Leu852 held the ligand through alkyl interactions (Table 3 and Figure 8B). The residues such as Lys753, Glu770, Met774, Leu796, Val797, Thr798, Gln799, Met801, Gly804, Val853, Thr862, Asp863, and Phe864 formed van der Waals interactions, positioning the ligand at the binding pocket (Table 3 and Figure 9B).

These results demonstrate that the compounds have been seated in the binding pocket of the target and might be useful in achieving the desired outcome. Interactions with these compounds were previously reported [62,63,64].

#### 2.6.7. Principal Component Analysis and Essential Dynamics

**Figure 10:**
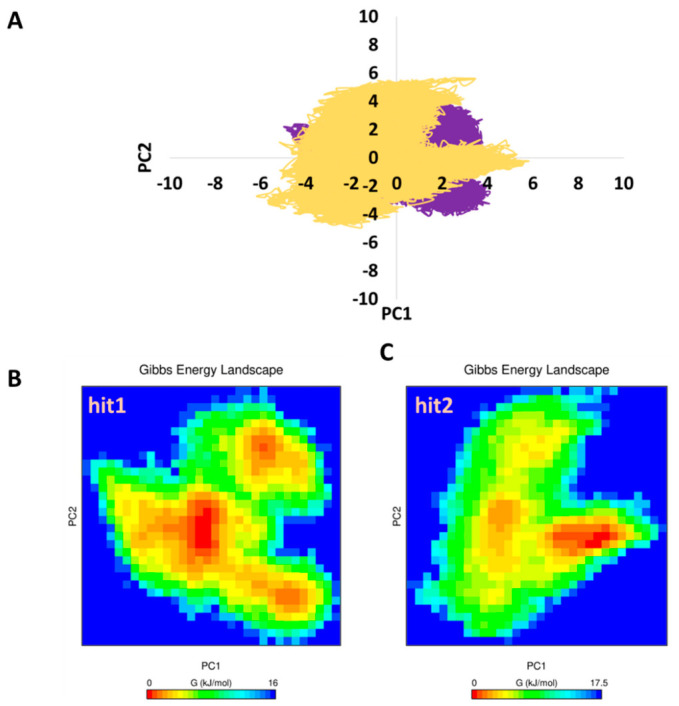
Essential dynamics results. (**A**) PCA of hit1 and hit2. (**B**) Gibbs free-energy landscape of hit1. (**C**) Gibbs free-energy landscape of hit2.

The principal component analysis can be utilized to probe into the conformational variations in the backbone of the protein [65]. Typically, a functional protein should be rigid while demonstrating flexibility, in particular with the residues at the binding site, which would permit good conformational sampling. The ED was undertaken to comprehend the overall protein movement in the conformational spaces with the help of PC1 and PC2 [66]. To determine the most significant subspace where the majority of protein dynamics take place, the covariance matrix of the eigenvectors was diagonalized to get PC1 and PC2. From the obtained results, it can be inferred that the motion of hit1 occurred in minor conformational spaces, while hit2 demonstrated navigation in PC2 to attain a stable complex (Figure 10A). The minimal-energy conformational ensembles of biomolecules are accurately described by the free-energy landscape. Furthermore, the ED results have demonstrated that hit1 and hit2 (Figure 10B,C) displayed two energy minima to obtain a stable state [66]. The Gibbs free-energy for hit1 ranged between 0 and 16 kJ/mol, and hit2 demonstrated a Gibbs free energy range between 0 and 17.5 kJ/mol, respectively, inferring that the hit1 complex was relatively more stable than hit2. Furthermore, the favorable and unfavorable conformations are represented in red and blue (Figure 10B,C).

## 3. Discussion

With an objective to retrieve novel natural compounds against HER2, the current investigation was undertaken employing various computational methods [67,68,69]. Reports exists that the computationally identified compounds have also been reported to show an in vitro or in vivo effect, illuminating their inhibitory potential [70,71,72]. In general, natural compounds demonstrate several therapeutic opportunities [73,74]. In the current investigation, pharmacophore based potential natural compounds were discovered against HER2 as plausible inhibitors using several computational techniques.

The two compounds have mapped to all the features of the pharmacophore model, thereby suggesting that the compound might show therapeutic potential (Supplementary Figure S3A,B). Furthermore, the hits have demonstrated a better molecular dock score than the inbound co-crystallized compound, implying their superiority as plausible and promising compounds. The MDS demonstrated that these compounds have maintained stable results, supported by key residue interactions. The key residues Leu726 and Val734 were observed with both hits, forming alkyl or/and π-alkyl interactions. Previous studies have also shown interaction with this residue [75,76,77]. Additionally, the residues Val797, Met801, Gly804, and Phe864 have rendered van der Waals interactions with both compounds. These interactions were also observed in the previous studies [62,78,79]. These findings elucidate that the identified compounds might be potential inhibitors against HER2. The ADMET predictions for both compounds show BBB as 2 (medium), a prediction that should be interpreted cautiously, since in silico BBB scores do not establish actual CNS exposure or therapeutic relevance to breast cancer brain metastases and would require dedicated pharmacokinetic evaluation [80].

These two compounds were retrieved from the CMNPD database with the IDs CMNPD30448 (hit1) and CMNPD7060 (hit2). The name of hit1 is Misszrtine A and it belongs to the *Fungi* Kingdom of *Aspergillus* sp. This marine sponge is an indole alkaloid [81,82]. This compound has exhibited potential anticancer activity, although, against several cancer cell lines. Interestingly, this compound has shown an IC_50_ >30 in SK-BR-3 cell lines [81]. This provides a biological precedent for bioactivity of this compound class; however, an IC50 >30 µM reflects only weak cytotoxic potency in a HER2-positive cell line and, on its own, does not establish HER2-specific inhibitory activity. The compound hit2 belongs to *Animalia* kingdom and *Lemnalia bournei* sp. and has not been tested against HER2.

We note two important limitations of the present study, which we make explicit here so that readers can correctly calibrate the strength of the computational evidence presented. First, compounds were classified as bioactive using an IC50 ≤ 1000 uM threshold. We recognize that this is markedly more permissive than the thresholds typically applied in ChEMBL-based bioactivity classification studies, which commonly use cutoffs in the 1–10 uM range [83]; a 1000 uM cutoff may therefore include compounds with only weak or marginal biological activity. This threshold was adopted specifically to retain a sufficiently large and class-balanced training set from the limited number of HER2 bioactivity records available in ChEMBL, a common trade-off when working with a single, moderately sized target dataset. We were not able to re-run the full descriptor-generation, model-training, and LIME-explanation pipeline at stricter thresholds (e.g., ≤10 uM or ≤1 uM) within the scope of the present study, and we did not have access to additional computational resources to do so during this revision. We therefore explicitly flag that the classification of CMNPD30448 and CMNPD7060 as ‘active’, and the reported model performance, are tied to this permissive threshold and have not been verified at stricter, more conventional cutoffs; this should be treated as an open question for follow-up work rather than a settled result. Second, the molecular dynamics analysis presented here (RMSD, Rg, RMSF, hydrogen-bond count, and visual binding-mode inspection over a single 500 ns trajectory per complex) demonstrates that the predicted hit1/hit2–HER2 complexes remain structurally stable over the simulated timeframe, but this does not by itself establish binding affinity or inhibitory potency. A more definitive assessment would require ligand RMSD after protein alignment, contact/hydrogen-bond occupancy analysis, MM/PBSA or MM/GBSA binding free-energy estimation, a benchmark simulation of the co-crystallized ligand or a known HER2 inhibitor under identical conditions, and independent simulation replicates to establish reproducibility. We were unable to perform these additional analyses within the scope of the present study, as they require new production simulations and post-processing beyond the computational resources available to us during this revision; we present them here as clearly identified, high-priority next steps rather than as results of the current work. Taken together, both points should be weighed when interpreting the strength of the computational evidence presented, and further in vitro and in vivo validation remains essential before any therapeutic claim can be made.

The current scenario in the CADD process involves the application of AI and ML models [57,84,85,86] along with XAI, that can help in understanding the models better in decision-making applications [55]. The XAI-based explanations are of two types: global and local [87]. In the current investigation, the random forest model has classified the two compounds as active, and LIME has put forth the features that contributed to this. The PubChem fingerprints that are present in hit1 are PubChem fingerprint440 [C(-C)(-O)(=O)], PubChem fingerprint452 [C(-O)(=O)], PubChem fingerprint380 [C(~O)(~O)], PubChem fingerprint566 [O-C-C-N] and PubChem fingerprint712 [C-C(C)-C(C)-C]. The hit2 has displayed fingerprints such as PubChem fingerprint700 [O-C-C-C-C-C-O-C], PubChem fingerprint380 [C(~O)(~O)], and PubChem fingerprint712 [C-C(C)-C(C)-C]. Taken together, we propose two compounds that might act as HER2 inhibitors.

## 4. Materials and Methods

### 4.1. Structure-Based Pharmacophore Generation

A structure-based pharmacophore approach exploits the critical features between the target and the ligand that are essential for therapeutic applications [88,89]. The target selected for the current investigation was the X-ray structure of the human HER2 kinase domain (PDB: 3PP0, resolution: 2.25 Å) with a co-crystallized ligand [42]. Furthermore, the Interaction Generation tool was enabled to gain insight into the pharmacophore features for all the residues around the co-crystallized ligand at 10 Å. Correspondingly, the pharmacophore models were generated by enabling the Receptor-Ligand Pharmacophore Generation protocol available in the DS. The parameters for maximum pharmacophores was selected as 10 with minimum features and maximum features as 4 and 5, respectively. Furthermore, the Rules selectivity scoring was used with a maximum charge distance, maximum hydrogen bond distance, and maximum hydrophobic distance set at 5.6, 3.0, and 5.5. The maximum exclusion volume distance was 2.0, with a minimum inter-feature distance of 2.0. The only projection point was chosen as False.

### 4.2. Pharmacophore Validation

Pharmacophore validation is an important step that assesses the ability of the pharmacophore to retrieve the active compounds from the given chemical databases [90,91]. Here, the decoy set validation was performed.

Validation of the pharmacophore model was conducted using the decoy set that demonstrates the usability of the pharmacophore model in distinguishing the active compounds from the inactive compounds [92]. The results of the decoy set are evaluated based on the enrichment factor (EF) and goodness of hit (GH). This method is sometimes referred to as the Güner–Henry scoring method [93]. Here, the number of compounds in the decoy set *D* is 1000, with a total of 90 active compounds *A*. Correspondingly, the GH and EF are calculated as follows:

$$EF=\left[\frac{\left(Ha\times D\right)}{\left(Ht\times A\right)}\right]$$

$$GH=\left[\left(\frac{Ha}{4HtA}\right)\left(3A+Ht\right)\times \left(1-\left(\frac{Ht-Ha}{D-A}\right)\right)\right]$$

In the above equation, *Ht* refers to the number of *hits* retrieved, and *Ha* refers to the total number of active compounds. In general, a GH score above 0.7 endorses that a pharmacophore model is good one [94].

### 4.3. Virtual Screening for the Retrieval of Drug-like Compounds

The well-validated pharmacophore model was employed to screen the chemical database with an objective of retrieving potential compounds against the target 3PP0. For the current investigation, the CMNPD database was used [95]. The CMNPD consists of manually curated compounds of marine natural products that comprise about 31,000 compounds. These compounds were downloaded in the .sdf format and upgraded to DS. Preparation of the compounds was accomplished by enabling the Full Minimization module by applying the CHARMm forcefield with the Smart Minimizer algorithm. For this, 1000 steps of the steepest descent were used, with an RMS gradient tolerance of 3. Thereon, the conjugate gradient minimization was applied. The so minimized compounds were upgraded for molecular docking.

The minimized compounds were subjected to absorption, distribution, metabolism, excretion, and toxicity (ADMET) using the ADMET Descriptors tool accessible with the DS. The selected upper limits for the parameters are absorption 0 (good), BBB 1, 2 (high, medium), solubility 2 (low), hepatotoxicity (false), and CYPD260 (false). The resultant compounds were scrutinized for Lipinski’s rule of five by applying the Filter by Lipinski and Veber Rule module with the upper limit set as the number of hydrogen bonds less than 10, the number of hydrogen bond donors less than 5, the molecular weight less than 500 Da, and the AlogP less than 5. Furthermore, the rotatable bonds were considered less than 10 and the polar surface area (TPSA) less than 140 ′Å2 [41,96]. Veber’s predictions ensure effective oral bioavailability [96].

The obtained compounds that satisfied the above mentioned criteria were upgraded to map the compounds from the database. The validated pharmacophore model was allowed to map the CMNPD, enabling the Ligand Pharmacophore Mapping protocol, opting for Fast for conformation generation and the Rigid fitting method with best mapping only as True.

### 4.4. Binding Affinity Analysis by Molecular Docking

Molecular docking studies are helpful in predicting the binding modes of the compounds or small molecules rendered by the molecular dock score. This also allows us to dislodge the false positive. Here, the LibDock available with the DS was used [97]. Diller and Merz engineered this molecular docking program that exploits the protein or target site features called HotSpots [98].

Prior to the initiation of the protocol, the target of interest (3PP0) was prepared by enabling the prepare protein tool available on the DS [59]. This protocol performs necessary functions that include inserting missing atoms in the residues that are incomplete [99,100], modeling the missing loops, removing the water molecules, omitting the alternate conformations, standardizing the atom names, protonating titratable residues by means of predicted pKs. The active site of the protein was selected for all the atoms around the co-crystallized ligand at 10.022 Å with the binding site XYZ coordinates of 16.6964 Å, 15.9962 Å, and 27.115 Å, respectively. Correspondingly, the active site residues are recorded as Leu726, GLy729, Val734, Ala751, Lys753, Glu770, Met774, Ser783, Leu785, Leu796, Thr798, Gln799, Leu800, Met801, Arg849, Asn850, Val851, Leu852, Thr862, Asn863, and Phe864. These docking parameters are initially validated by redocking the co-crystallized ligand into the binding site (Supplementary Figure S2A,B). The result has shown that the redocked pose was also similar to that of the co-crystallized ligand, with an acceptable RMSD of 0.95 Å. The co-crystallized ligand is employed as the reference compound during the molecular docking studies. The prepared target and the ligands were subjected to molecular docking, allowing 100 conformations to be generated. The best pose was selected from the largest cluster with a higher LibDock score than the reference ligand and key residue interactions. The higher the LibDock score, the greater the protein–ligand interaction [101].

### 4.5. Predicting the Compounds Bioactivity

The compounds with the highest dock score are subjected to artificial intelligence and machine learning methods to predict if they are active or inactive. This study was undertaken considering the *PaDEL* fingerprints [43] for the known compounds with biological activity downloaded from ChEMBL [102] with the target query ‘HER2’ with bioactivity unit IC_50_ ‘uM’. Furthermore, the ‘null values’ and duplicate ‘SMILES’ were deleted. The resultant compounds were categorized as active (IC_50_ ≤ 1000, 0) and the remaining as inactive (1), and they were subjected to PaDEL fingerprints [43] generation using Padelpy in Google Colab. To obtain the dynamic results, various fingerprints were calculated, such as Molecular ACCess Systems (MACCS), Electrotopological State (Estate), AtomPairs2D, PubChem, and Substructure fingerprints. The data was split into training (80) and test (20) in the ratio of 80:20 and normalized with StandardScaler [103]. The classification models were random forest, XGBoost, KNN, logistic regression, decision tree, and ANN [104]. These are implemented on python-based scikit-learn and the relevant packages while the ANN was implemented on the TensorFlow platform with the neural network Application Programming Interface (API) Keras. The model with highest accuracy was saved using ‘pickle’ to predict the activity of new compounds.

The random forest method is a popular approach for a regression and classification method. This algorithm was initiated by L. Breiman. Here, multiple randomized decision trees are combined and averaged to provide an overall prediction [105]. The parameters selected were n_estimators as 100, criterion as gini, and random_state as 10. The eXtreme Gradient Boosting (XGBoost) is a robust machine learning approach that employs tree boosting to avoid overfitting [106] and is one of the best approaches for classification models. For the current study, we have used random_state as 0. The k-Nearest Neighbors (kNN) is an elegant approach performed for classification [107]. This method is significantly employed for data mining activities [108]. Here, the n_neighbors was taken as 5. Logistic regression is typically used to evaluate the correlation between the independent variable and the binary dependent variable. This technique is a broadened method of linear regression [109]. The decision tree classification is a tree-based approach that starts from a root to a leaf by correspondingly splitting the data and continues until a result at the leaf node is obtained [110]. This ensures a superlative methodology for data classification [111]. For the current investigation, the parameters used are criterion as gini and random_state as 1. Artificial Neural Networks (ANN) depict the work like that of biological neurons that consist of layers typically labeled as the input layer, hidden layer(s), and output layer [112]. These have become beneficial strategies for simulating nonlinear structure–activity relationships [113]. For the current investigation, we have used an input layer, a hidden layer with activation as ReLU [114], and an output layer with activation as sigmoid. The model was complied with loss as binary_crossentropy, an optimizer as adam, and metrics as accuracy. The model was fit with 200 epochs and a batch size of 10. The best poses from the molecular docking results that are predicted as active compounds from AI/ML methods were escalated to molecular dynamics simulation studies for in-depth analysis of the target-ligand complex.

#### 4.5.1. Evaluation Metrics

The quality and robustness of the ML models is assessed by evaluation metrics. For the current investigation, the accuracy, precision, recall, F1-score and receiver operating characteristic area under the curve (ROC AUC) are used. These are obtained from a confusion matrix.

#### 4.5.2. Confusion Matrix

In the classification models, one method of evaluating the model’s robustness is to know the number data points that are classified accurately and inaccurately. Correspondingly, it is essential to understand the class of data that is wrongly classified. One such approach is the confusion matrix that assists in understanding this problem. A confusion matrix is a matrix that illustrates the degree at which a machine learning model operates on a set of data. This is a two-dimensional matrix with rows and columns in which the rows encode the actual (true) classifications and the columns denote the predicted classifications. In the event that the model executes seamlessly, scores will only display in diagonal places. Otherwise, if there are any wrong predictions these are placed in the off-diagonal position. Thus a confusion matrix is simple, elegant, and yet a powerful method to discover which classes have been predicted wrongly [115,116].

Depending on the model’s predictions, it distinguishes the accurate data from the inaccurate data according to true positive (TP), true negative (TN), false positive (FP), and false negative (FN), (Table 4).

True Positive (TP): This indicates that the model has correctly determined the positive outcome (the actual label is positive, predicted as positive).

True Negative (TN): This indicates that the model has correctly determined the negative outcome (the actual label is negative, predicted as negative).

False Positive (FP): In this case, the models predicts the outcome as positive for an actual negative label (the actual label is negative, predicted as positive). This is also called a Type I error.

False Negative (FN): In this case, the models predicts the outcome as negative for an actual positive label (the actual label is positive, predicted as negative). This is also called a Type II error.

Accuracy: The accuracy of a model is a criteria to estimate the proportion of accurate predictions to all data assessed. This is the proportion of correctly classified instances among all evaluated instances, calculated as Accuracy = (TP + TN)/(TP + TN + FP + FN). A good prediction refers to high accuracy and high precision [117].

Accuracy=TP+TN/TP+TN+FP+FN

Precision: Precision provides the model’s positive prediction accuracy. Precision determines the degree of correctness of the model’s prediction. In simple terms, precision accounts to the number of accurate data points obtained divided by the total number of the data points obtained [117].

Precision=TP/TP+FP

Recall: This is the ratio of proportion of the number of real positive predictions as identified by the model to the number of actual positive predictions in a given data. This determines the effectiveness of positive prediction. Typically, this feature accounts to the number of correct data points divided by all the correct data points [117].

Recall=TP/TP+FN

F1-Score: The F-score (F1-score, F-measure) is the weighted average of precision and recall depending on the weight function β and is known as the F-score. The harmonic mean of precision and recall is known as the F1-score. Different indices can be used to calculate the F1-score, attributing varying weights to recall and precision [117].

$$F1score=2\times (\frac{Precision\times Sensitivity}{Precision+Sensitivity})$$

#### 4.5.3. Area Under the ROC Curve (AUC)

This is one of the superlative methods that is adapted to construct an enhanced learning model and to compare the learning algorithms. Correspondingly, this method mirrors the comprehensive ranking ability of the model and has been established as a better metric than the accuracy. However, the computational cost seems to be greater for multiclass problems [118]. Typically, ROC is a probability curve and AUC demonstrates the distinguishing ability between the labels, which implies that higher the AUC, the better is the model’s performance [119].

### 4.6. Molecular Dynamics Simulation Studies

The behavior of small molecules at the binding pocket of the target protein is deciphered using molecular dynamics simulations (MDS) using GROMACS v2016.6 [120]. The main purpose of the MDS is to assess the protein–ligand binding stability and to comprehend the interactions at the atomistic level. Accordingly, the ligand topologies were obtained from SwissParam [121], employing the CHARMM27 all-atom force field. Following this, a dodecahedron water box was built and solvated with the TIP3P water model. Correspondingly, the counter ions (Cl^−^) were added, and the system was minimized. The two-step equilibration was conducted after the coupling of the protein and ligand. The first step of equilibration was executed with a conserved number of particles (N), system volume (V), and temperature (T) (NVT) for 1 ns, and the second equilibration step was done with a conserved number of particles (N), system pressure (P), and temperature (T) (NPT) for 1 ns with a V-rescale thermostat. The 500 ns was applied to the NPT. The backbone was restrained during equilibration, and the pressure was maintained with a Parrinello–Rahman barostat [122]. The geometry of the molecules was monitored with the LINCS algorithm [123], and Particle Mesh Ewald (PME) [124] was used to calculate the long-range electrostatic interactions. The obtained results were evaluated with visual molecular dynamics (VMD) [125] and DS for the backbone of the protein. The results were elucidated based on root mean square deviation (RMSD), radius of gyration (Rg), root mean square fluctuations (RMSF), hydrogen-bond count, and the binding mode analysis of the ligand at the binding pocket. In addition, the gmx covar and gmx sham were used to study the principal component analysis (PCA) and essential dynamics (ED) to comprehend the dynamic movement of the protein.

## 5. Conclusions

With an aim to retrieve potential compounds against HER2, the current investigation proceeded employing numerous computational tools. Here, the structure-based pharmacophore model was built to retrieve compounds from CMNPD. These compounds have displayed favorable molecular docking results and molecular dynamics simulations. Furthermore, using different ML methods were used on various fingerprints to classify these novel compounds as ‘active’. The important fingerprints that have contributed to ‘active’ were recommended by explainable LIME. Taken together, we propose the two compounds CMNPD30448 (hit1) and CMNPD7060 (hit2) that might act as plausible HER2 inhibitors.

## Figures and Tables

**Table 1:** LibDock docking scores of the reference co-crystallized ligand and the identified hit compounds.

Compound	LibDock Score	Description
Reference compound	152.553	Co-crystallized ligand (PDB: 3PP0)
CMNPD30448 (hit1)	164.72	Retrieved marine natural product hit
CMNPD7060 (hit2)	158.332	Retrieved marine natural product hit

**Table 2:** Evaluation metrics results of various ML methods for different FPs.

**FP/Models**	**MACCS**							
**Evaluation Metrics**	**Accuracy**	**Precision**		**Recall**		**F1-Score**		**ROC-AUC**
		**0**	**1**	**0**	**1**	**0**	**1**	
Random Forest	0.87	0.84	0.90	0.90	0.84	0.87	0.87	0.94
XGBoost	0.51	0.00	0.51	0.00	1.00	0.00	0.68	0.68
KNeighborsClassifier	0.80	0.77	0.85	0.86	0.75	0.81	0.80	0.90
Logistic Regression	0.84	0.85	0.84	0.83	0.86	0.84	0.85	0.89
Decision Tree	0.86	0.85	0.88	0.88	0.85	0.86	0.86	0.86
Artificial Neural Network	0.86	0.85	0.88	0.88	0.85	0.86	0.86	0.86
**FP/Models**	**Atompairs2D**							
**Evaluation Metrics**	**Accuracy**	**Precision**		**Recall**		**F1-Score**		**ROC-AUC**
		**0**	**1**	**0**	**1**	**0**	**1**	
Random Forest	0.84	0.84	0.84	0.83	0.85	0.84	0.85	0.93
XGBoost	0.52	0.67	0.51	0.01	0.99	0.03	0.68	0.72
KNeighborsClassifier	0.79	0.77	0.81	0.81	0.78	0.79	0.79	0.86
Logistic Regression	0.82	0.83	0.81	0.79	0.85	0.81	0.83	0.88
Decision Tree	0.81	0.77	0.85	0.86	0.76	0.81	0.80	0.81
Artificial Neural Network	0.82	0.82	0.8	0.8	0.84	0.82	0.83	0.82
**FP/Models**	**Estate**							
**Evaluation Metrics**	**Accuracy**	**Precision**		**Recall**		**F1-Score**		**ROC-AUC**
		**0**	**1**	**0**	**1**	**0**	**1**	
Random Forest	0.86	0.85	0.88	0.89	0.84	0.87	0.86	0.92
XGBoost	0.49	0.25	0.49	0.01	0.98	0.01	0.65	0.70
KNeighborsClassifier	0.81	0.79	0.85	0.87	0.76	0.83	0.80	0.88
Logistic Regression	0.80	0.77	0.83	0.85	0.75	0.81	0.79	0.85
Decision Tree	0.84	0.80	0.88	0.89	0.78	0.85	0.82	0.86
Artificial Neural Network	0.84	0.80	0.88	0.89	0.78	0.85	0.82	0.83
**FP/Models**	**Substructure**							
**Evaluation Metrics**	**Accuracy**	**Precision**		**Recall**		**F1-Score**		**ROC-AUC**
		**0**	**1**	**0**	**1**	**0**	**1**	
Random Forest	0.85	0.81	0.90	0.91	0.80	0.86	0.85	0.93
XGBoost	0.51	0.25	0.51	0.01	0.98	0.01	0.67	0.47
KNeighborsClassifier	0.81	0.77	0.86	0.87	0.76	0.82	0.80	0.87
Logistic Regression	0.81	0.79	0.83	0.83	0.78	0.81	0.81	0.87
Decision Tree	0.83	0.78	0.89	0.90	0.76	0.84	0.82	0.85
Artificial Neural Network	0.85	0.84	0.86	0.86	0.84	0.85	0.85	0.84
**FP/Models**	**PubChem**							
**Evaluation Metrics**	**Accuracy**	**Precision**		**Recall**		**F1-Score**		**ROC-AUC**
		**0**	**1**	**0**	**1**	**0**	**1**	
Random Forest	0.91	0.92	0.90	0.90	0.92	0.91	0.91	0.96
XGBoost	0.51	0.00	0.51	0.00	1.00	0.00	0.68	0.71
KNeighborsClassifier	0.86	0.83	0.89	0.90	0.83	0.86	0.86	0.9
Logistic Regression	0.87	0.87	0.87	0.86	0.86	0.87	0.87	0.92
Decision Tree	0.86	0.85	0.87	0.86	0.86	0.86	0.86	0.85
Artificial Neural Network	0.88	0.88	0.88	0.87	0.89	0.88	0.88	0.87

**Table 3:** Comprehensive intermolecular interactions between the hits and the key target residues.

Compound Name	LibDock Score	Hydrogen Bond	Alkyl/π-Alkyl Interactions	Van der Waals Interactions
CMNPD30448 (hit1)	164.72	Ala751 and Thr862	Leu726, Val734, Leu796, and Leu852	Gly727, Ser728, Gly729, Ile752, Lys753, Met774, Ala775, Val797, Leu800, Met801, Gly804, Arg849, Asn850, Asp863, and Phe864
CMNPD7060 (hit2)	158.332	Ser783	Leu726, Val734, Ala751, Leu800, Cys805, and Leu852	Lys753, Glu770, Met774, Leu796, Val797, Thr798, Gln799, Met801, Gly804, Val853, Thr862, Asp863, and Phe864

**Table 4:** Confusion matrix framework.

		Predicted Values	
		Predicted Positive	Predicted Negative
Actual Values	Actual Positive	TP (The actual label is positive, predicted as positive)	FN (The actual label is positive, predicted as negative)
	Actual Negative	FP (The actual label is negative, predicted as positive)	TN (The actual label is negative, predicted as negative)

## Data Availability

The original data presented in the study are openly available in GitHub at https://github.com/SRampogu/HER2 (accessed on 12 March 2026).

## Supplementary Materials

The following supporting information can be downloaded at: https://www.mdpi.com/article/10.3390/ijms27146504/s1.
