# Supplementary material for: Explainable Artificial Intelligence (XAI) and Molecular Modeling Techniques to Discover Putative HER2 Inhibitors
Source: Int J Mol Sci. 2026 Jul 22;27(14):6504. doi: 10.3390/ijms27146504 (PMC13411091; doi:10.3390/ijms27146504)
Supplement: Supplementary file 1 [file ijms-27-06504-s001.zip › Supplementary_Table.pdf]

Supplementary Table S1

Pharmacophore Summary			
Pharmacophore	Number of Features	Feature Set	Selectivity Score
Pharmacophore_01	5	ADHHH	9.4812
Pharmacophore_02	5	ADHHH	9.4812
Pharmacophore_03	5	ADHHH	9.4812
Pharmacophore_04	5	ADHHH	9.4812
Pharmacophore_05	5	ADHHH	9.4812
Pharmacophore_06	5	ADHHH	9.4812
Pharmacophore_07	5	AADHH	9.4812
Pharmacophore_08	5	ADHHH	9.4812
Pharmacophore_09	5	AADHH	9.4812
Pharmacophore_10	5	DHHHH	9.4812
