# Supplementary material for: Explainable Artificial Intelligence (XAI) and Molecular Modeling Techniques to Discover Putative HER2 Inhibitors
Source: Int J Mol Sci. 2026 Jul 22;27(14):6504. doi: 10.3390/ijms27146504 (PMC13411091; doi:10.3390/ijms27146504)
Supplement: Supplementary file 1 [file ijms-27-06504-s001.zip › Supplementary_Figures.pdf]

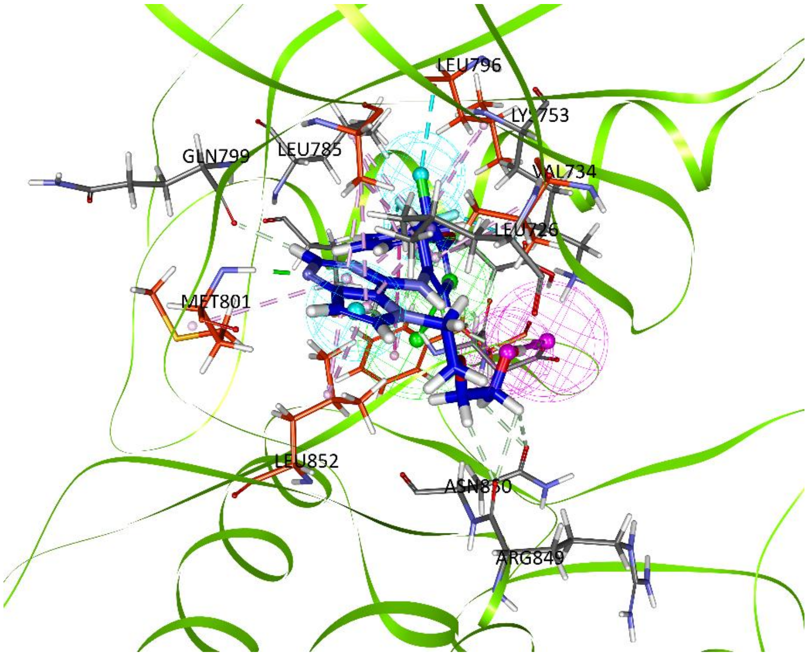

Supplementary Figure S1. Key residues aligned with the pharmacophore model

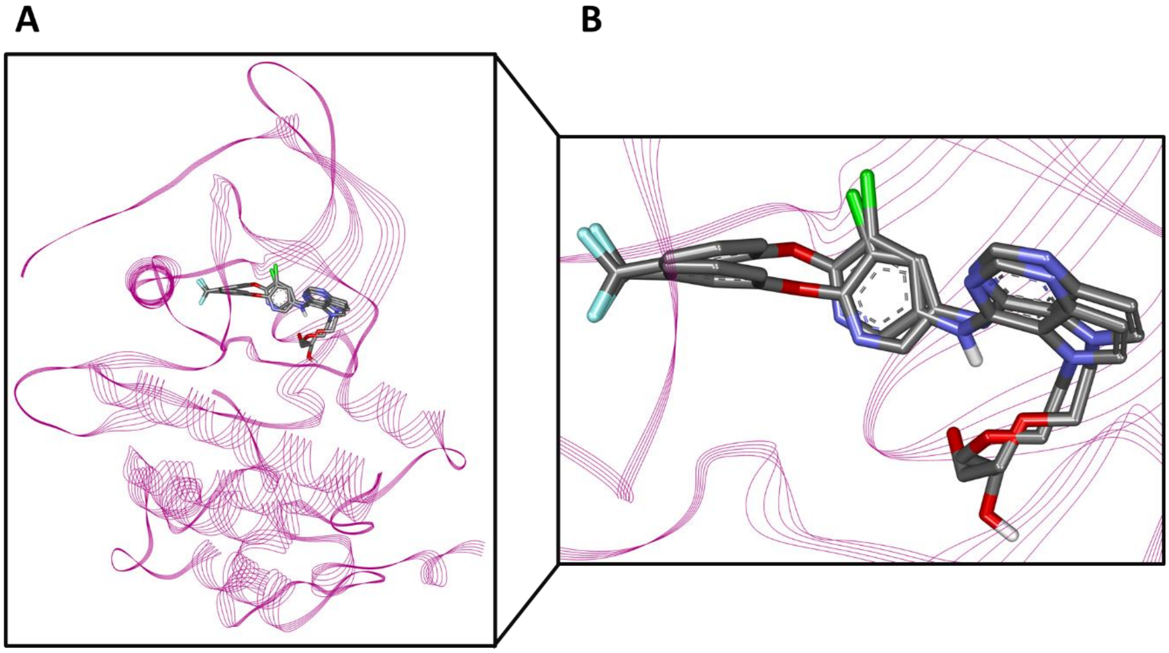

Supplementary Figure S2. Molecular redocking of cocrystalling ligand. The result shows that the redocked ligand has demonstrated a similar binding pose as that of the inbound ligand. 2A) overlay of inbound ligand and the molecular docked pose. 2B) The zoomed version of the ligands.

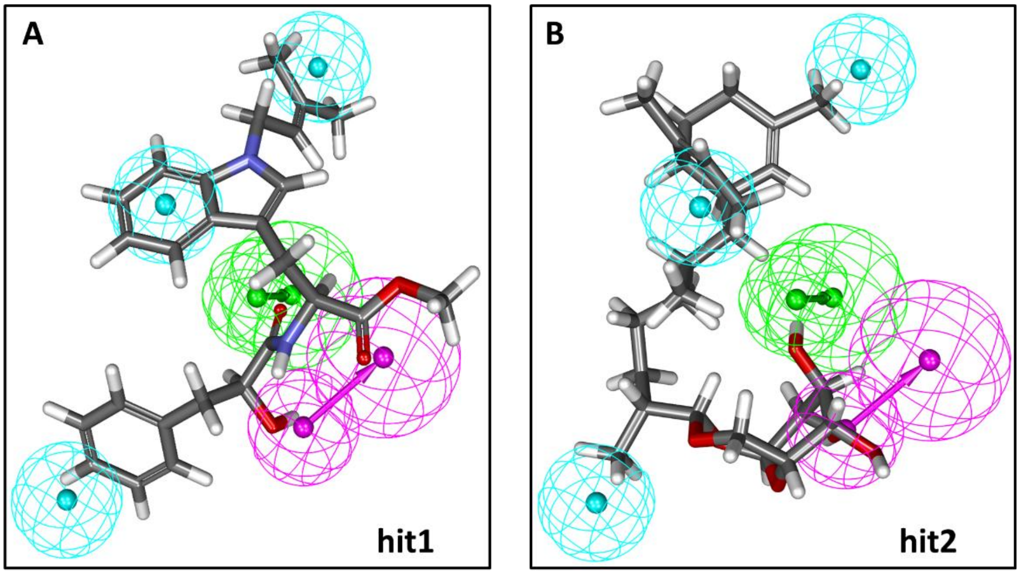

Supplementary Figure S3. The retrieved hits map to all the features of the pharmacophore model. 3A) Pharmacophore mapping to hit1. 3B) Pharmacophore mapping to hit2.
